# Tuning Competing Electronic Phases in Monolayer VSe_2_ via Interface Hybridization

**DOI:** 10.1021/acsnano.6c04065

**Published:** 2026-07-24

**Authors:** Ishita Pushkarna, Árpád Pásztor, Greta Lupi, Adolfo O. Fumega, Christoph Renner

**Affiliations:** † Department of Quantum Matter Physics, 27212 University of Geneva, 1211 Geneva, Switzerland; ‡ Department of Applied Physics, 174277 Aalto University, 02150 Espoo, Finland

**Keywords:** charge density waves, interface hybridization, VSe_2_ on gold, heterostructure, moiré, ab initio methods

## Abstract

Competing electronic phases in two-dimensional transition metal dichalcogenides constitute a fertile platform for uncovering emergent ground states and elucidating the control parameters that govern the correlated electron phases. Among these materials, vanadium diselenide is particularly compelling: while the bulk hosts a well-established charge density wave (CDW), monolayers exhibit markedly different electronic behavior. Here, we identify three distinct electronic regimes in mechanically exfoliated VSe_2_ flakes on Au(111) substrates, where interfacial hybridization, charge transfer, and strain act as primary tuning parameters of electronic order. Monolayers strongly coupled to gold show complete suppression of the CDW, accompanied by the emergence of moiré modulations. In contrast, bilayers preserve the in-plane 4*a* × 4*a* CDW characteristic of the bulk limit. Strained, electronically decoupled monolayers formed in suspended membrane and bubble regions stabilize a √3*a* × √7*a* CDW phase, underscoring the reversible role of substrate interaction and hybridization.

## Graphical abstract

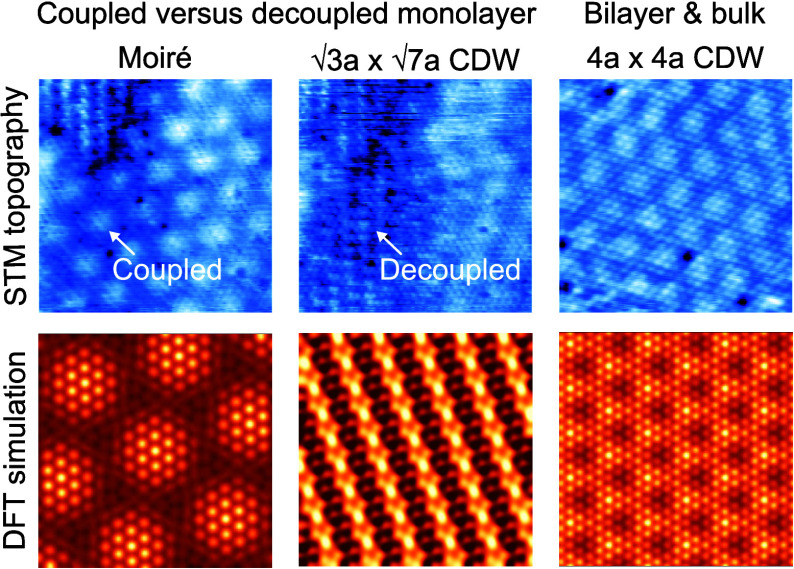

Competing electronic phases and their tunability are of central interest in contemporary solid-state physics. Traditional tuning parameters include chemical doping, hydrostatic pressure, strain, and magnetic field. With the emergence of two-dimensional (2D) materials, new possibilities appear, such as dimensionality,^1,2^ electrostatic gating,^3–5^ and controlled stacking into heterostructures enabling proximity effects, moiré physics, and interface engineering.^6–8^ VSe_2_ is particularly interesting in this context. It is a metallic transition-metal dichalcogenide (TMD) which, in its bulk form, hosts a unique 4*a* × 4*a* × 3.2*c* (where *a* and *c* are the in-plane and out-of-plane lattice constants, respectively) charge density wave (CDW) below *T*_c_ = 105 K.^9–11^ This CDW can be altered by V self-intercalation,^12^ by pressure,^13^ and in-plane strain.^14^ The CDW transition temperature has been found to depend non-monotonically on thickness.^1,15,16^

VSe_2_ is among the most three-dimensional TMDs, with sizable interlayer interaction resulting in significant dispersion in the energy spectrum along *k*_*z*_,^17,18^ making it notoriously difficult to exfoliate. Investigating monolayer (ML) VSe_2_ has thus relied on molecular beam epitaxy (MBE).^19–33^ Interest in this compound is primarily fueled by the ferromagnetic (FM) order predicted by theory in ML VSe_2_.^34–36^ Following the first experimental observation of strong room temperature ferromagnetism in ML VSe_2_,^19^ several subsequent studies reported only weak^24,29,30^ or no magnetic signal^20,22,23^ in these MBE-grown samples, independently of the substrates. Theory has shown that the CDW plays a crucial role in the magnetic properties of ML VSe_2_, with competition from the CDW suppressing ferromagnetic ordering.^37,38^ However, observations on MBE-grown ML VSe_2_ are widely scattered regarding magnetism and charge order. Reported CDW transition temperatures (*T*_CDW_) range from 350 K (ML grown on bilayer graphene on SiC substrate (BLG-SiC))^24^ to no transition at all (ML grown on NbSe_2_ substrate).^30^ Such widely varying behavior is even reported for ML grown on the same substrate. For example, very different *T*_CDW_ of 140 K,^20,22^ 171 K,^29^ 220 K,^29^ and 350 K^24^ have been reported for ML VSe_2_ on BLG-SiC.

Likewise, the reported energy gap in the quasi-particle spectrum associated with the CDW ground state ranges between 9 meV^24^ and 230 meV^22^ for ML VSe_2_ on BLG-SiC. The same variability has been observed on other substrates such as highly ordered pyrolytic graphite or MoS_2_. Finally, there is no consensus on the real-space structure of the CDW either. Although theoretical works support the possibility of different CDW reconstructions subject to strain, doping, and interaction with the substrate,^39,40^ the variability of the experimentally observed charge modulations (even on the same substrates) is astonishing: 3.2*a* × 2.24*a*,^19^ 4*a* × 4*a*,^20,22^ √3*a* × 2*a*,^24,26^ √3*a* × √7*a*,^21,23,24,26^ 4.2*a* × 4.6*a* together with a 2.33*a* stripe phase,^29^ and two simultaneous stripe phases with 4*a* and 2.8*a* periodicity^32^ have been reported.

It has been demonstrated that in TMDs such as NbSe_2_,^41^ TaS_2_,^42^ and IrTe_2_,^43^ interfacing monolayers with Au(111) is an effective strategy to suppress electronic instabilities like CDWs and superconductivity. The suppression arises from hybridization with the substrate, leading to pseudodoping and a reduction of the effective coupling constants.^44^ Moreover, the interaction strength with the substrate depends on the rotational alignment of the constituents,^6,45^ potentially offering a new means of tunability.

Here, we investigate monolayer VSe_2_ exfoliated on Au(111) using scanning tunneling microscopy and spectroscopy (STM/STS), with a particular emphasis on the effect of interface coupling on the CDW. Bilayer (BL) and thicker VSe_2_ flakes host a 4*a* × 4*a* CDW. Monolayer flakes host a different √3*a* × √7*a* CDW, but only when they are decoupled from the gold substrate. The CDW is absent in coupled ML, where STM topography reveals a moiré pattern consistent with the twist angle. DFT calculations confirm the formation of different CDW patterns in decoupled ML and BL VSe_2_ and its suppression when the ML is coupled to the gold substrate.

## Results and Discussion

We prepared ML flakes by exfoliating bulk crystals onto template-stripped gold substrates^6^ that consist in a mosaic of (111) oriented grains (see Methods section). This process yields a large area predominantly covered with macroscopic ML islands and occasional smaller BL regions revealed by STM in Figure 1(a) (also shown in Supporting Figure 1). The exfoliated MLs closely follow the gold steps of the substrate and are identified by their apparent height of 8 ± 4 Å relative to the Au(111) surface. The BLs appear consistently higher by 6 ± 0.5 Å (Figure 1(a) inset), corresponding to the interlayer spacing of bulk VSe_2_.^46^ From a theoretical point of view, free-standing monolayer 1T-VSe_2_ in its normal state (NS) (Figure 1(b)) is a highly unstable phase at low temperatures. The calculated electronic density of states (DOS), shown in Figure 1(c), reflects the unstable metallic character of the system, with partially occupied V *d*-orbitals and an enhanced density of states in the vicinity of the Fermi level, consistent with previous studies of monolayer VSe_2_.^19,20,35,38,40,47^ The combination of this enhanced low-energy density of states and electron–phonon coupling promotes the emergence of a CDW instability at low temperatures.^40,48,49^ This can be analyzed through the phonon spectrum in Figure 1(d), where it can be seen that free-standing monolayer VSe_2_ displays dynamical instabilities leading to the formation of different CDWs. Specifically, at $3/5\overset{®}{\Gamma K}$ and $1/2\overset{®}{\Gamma M}$ points associated with a √3*a* × √7*a* CDW and a 4*a* × 4*a* CDW, respectively. The √3*a* × √7*a* reconstruction originates from a phonon instability at a general wave vector along Γ–K, which connects high-density regions of the Fermi surface and leads to a rotated commensurate superstructure. Anharmonic calculations have shown that √3*a* × √7*a* CDW dominates in the tensile strain regime, while the 4*a* × 4*a* CDW occurs in the unstrained and compressive scenarios.^40^

**Figure 1:**
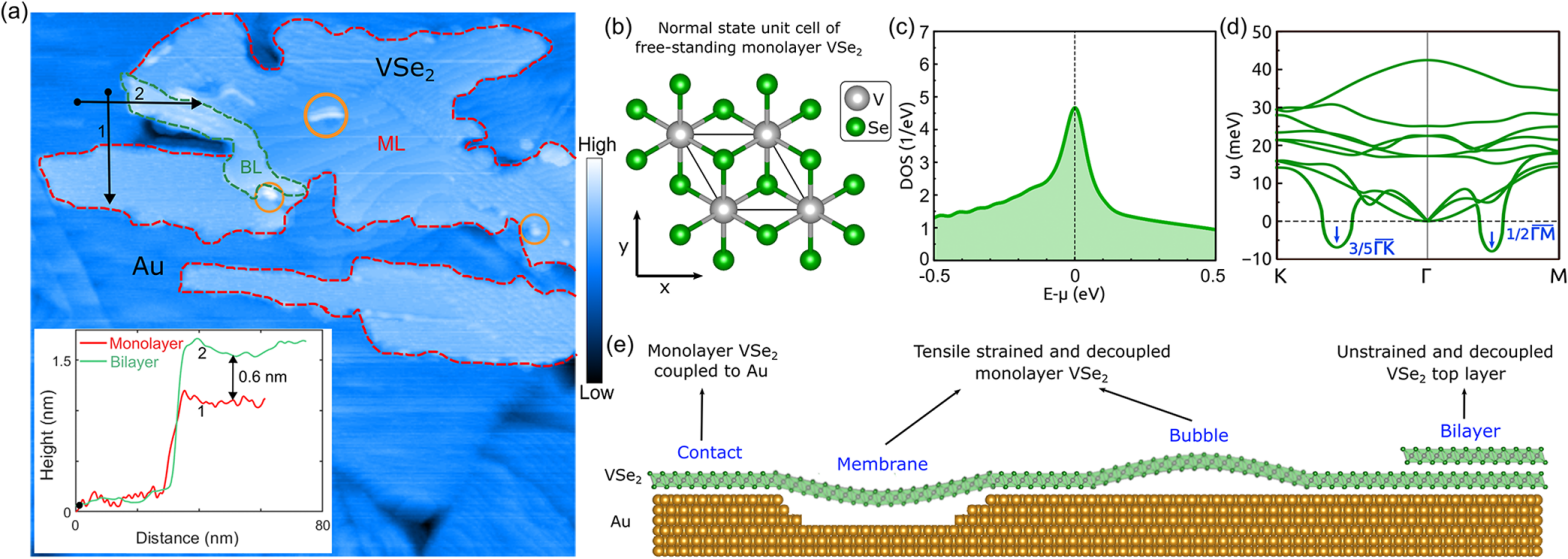
(a) STM topography (350 × 350 nm^2^, *V*_bias_ = 500 mV, *I*_t_ = 20 pA) of exfoliated VSe_2_ flakes on a Au(111) mosaic substrate, with the inset showing line profiles taken over ML and BL flakes. ML flakes are highlighted with red contours, BL flake is highlighted with green contour, and selected decoupled bubbles are circled in orange. (b) Normal state unit cell of free-standing monolayer VSe_2_. (c) Density of states (DOS) for free-standing or decoupled monolayer VSe_2_ in the normal state without CDW. (d) Harmonic phonon calculations for free-standing monolayer VSe_2_. (e) Schematic of the sample with ML and BL flakes of VSe_2_ (green) on gold (yellow); and possible decoupling scenarios: membrane over a pit in the gold or bubbles due to trapped material. Note that membranes and bubbles are subject to tensile strain.

The situation changes markedly when the ML is exfoliated on the Au(111) substrate. Our sample preparation offers unique opportunities to assess the influence of the substrate on the VSe_2_ flakes. We identify three distinct configurations (see Figure 1(a) and Supporting Information Section I): (i) monolayer VSe_2_ on Au(111) (red outline), (ii) bilayer VSe_2_ on Au(111) (green outline), and (iii) bubbles (orange circles) and membranes of monolayer VSe_2_. Each of them is schematically illustrated in Figure 1(e), and will be characterized in the following.

### Monolayer and Bilayer VSe_2_ on Au(111)

Atomic-resolution STM images of all bilayer flakes reveal a triangular atomic lattice accompanied by a perfectly commensurate, albeit weak, 4*a* × 4*a* periodic modulation (Figure 2(a)), reminiscent of the CDW observed in bulk (Figure 2(c)) and thin flakes.^1^ When the STM tip is scanned over a monolayer region, the topography reveals a periodic charge modulation whose periodicity and orientation depend on the local twist angle between VSe_2_ and the Au(111) substrate (see Figure 3), consistent with a moiré pattern, instead of the commensurate 4*a* × 4*a* CDW modulation observed when scanning the adjacent BL (Figure 2(b)). These patterns are consistent with moiré structures generated between rotated Au(111) grains and MLs obtained from a single crystal exfoliation oriented along the same direction highlighted by the yellow-dotted lines in Figure 3. At the largest twist angle between VSe_2_ and Au(111) in Figure 3(d), the moiré pattern is more challenging to identify. The assignment of the 4*a* × 4*a* CDW in the BL flakes and the moiré patterns in the ML flakes is further corroborated by their different bias-dependencies (contrast inversion versus no contrast inversion^50^) and by their characteristic behavior near defects (weak pinning of the CDW in VSe_2_ ^51,52^ versus no influence for the moiré), as described in Sections II and III of the Supporting Information. Note that coupled MLs never show any CDW modulation, and we do not observe a systematic variation in the spectroscopy, although the hybridization strength can depend on twist angle.^6,45^

**Figure 2:**
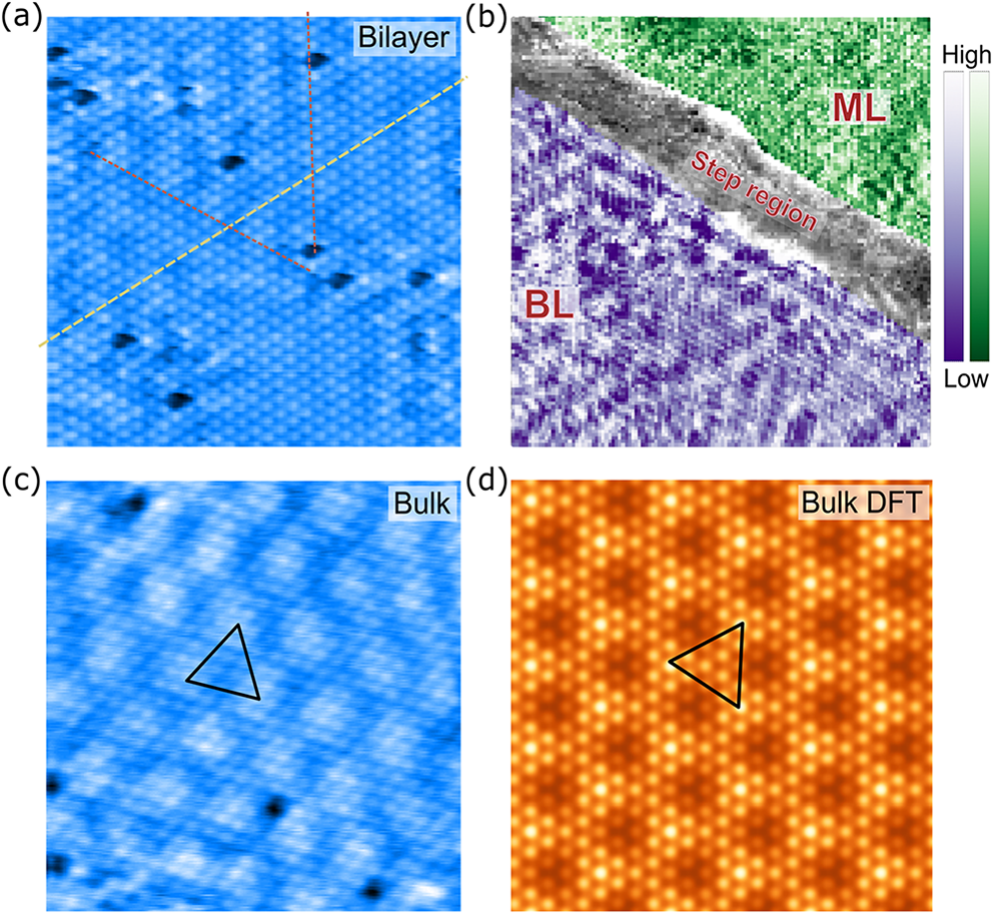
(a) STM topography (9 × 9 nm^2^, *V*_bias_ = 200 mV, *I*_t_ = 200 pA) on a BL region showing a weak 4*a* × 4*a* CDW modulation along with the atomic lattice. The red and yellow dashed lines mark the CDW and atomic directions, respectively. (b) Local DOS map (at *V*_bias_ = −70 meV) over the boundary (gray) between a BL (purple) and ML (green) region. (c) STM topography (8 × 8 nm^2^, −100 mV, 200 pA) on bulk VSe_2_ showing the in-plane 4*a* × 4*a* CDW modulation (black triangle) along with the atomic lattice. (d) DFT simulated image of the 4*a* × 4*a* CDW modulation.

**Figure 3:**
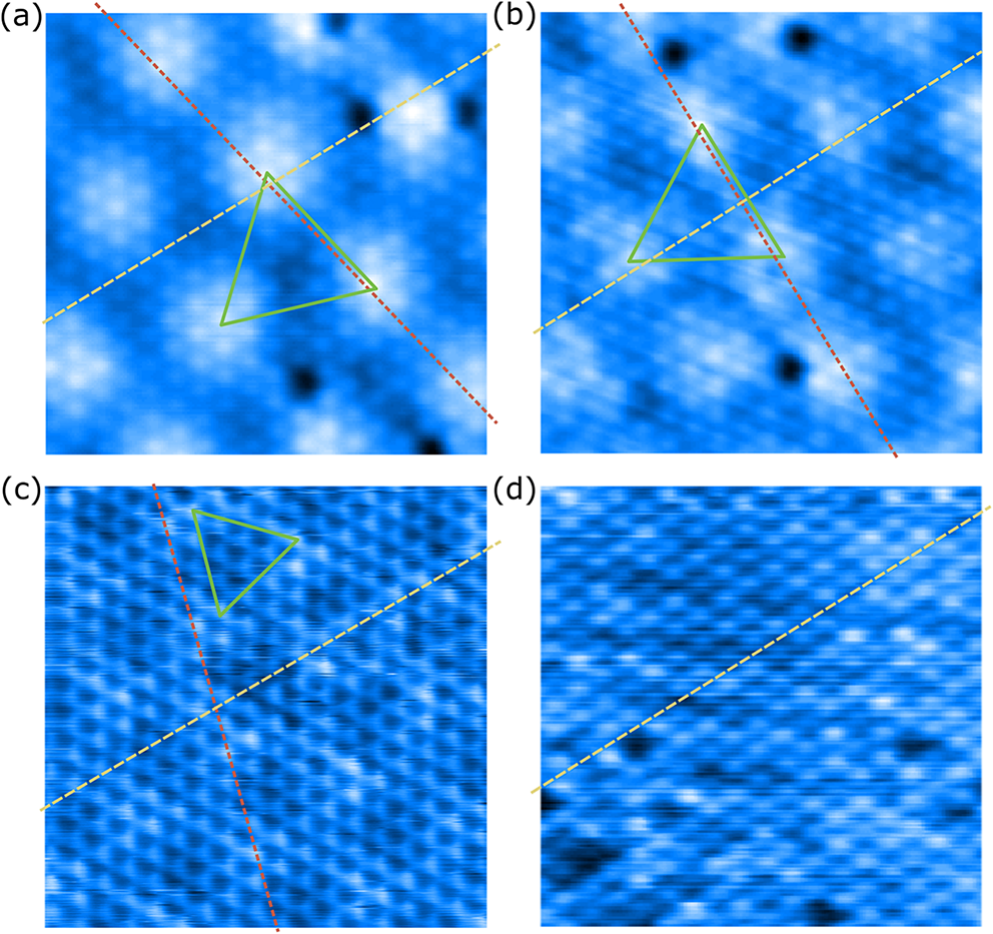
STM topography (5 × 5 nm^2^) of an exfoliated VSe_2_ ML on a mosaic Au(111) substrate. The red and yellow dashed lines mark the moiré and atomic lattice directions, respectively, and the green triangles denote the moiré superstructure. Note the same atomic lattice orientation in all four images, as expected for flakes originating from the same single crystal. The imaging bias and current setting for the different scans are (a) *V*_bia*s*_ = −100 mV, *I*_t_ = 200 pA, (b) 300 mV, 200 pA, (c) 500 mV, 100 pA, (d) 150 mV, 500 pA.

From a theoretical point of view, the 4*a* × 4*a* reconstruction observed in the bilayer region is in agreement with DFT simulations of the 4*a* × 4*a* CDW. For the monolayer in contact with gold, ab initio calculations reveal a strong hybridization between VSe_2_ states and the Au(111) substrate, which modifies the electronic spectrum, as discussed below. The interaction with Au(111) induces a pseudodoping effect.^44^

The hybridization reshapes the electronic bands in such a way that V *d*-states appear as heavily electron-doped relative to the free-standing case. This electronic reconstruction has direct consequences for the lattice dynamics, inducing a stabilization of the normal state and quenching the CDW phases. Simulated STM images for the coupled configuration exhibit a markedly different contrast compared to the bilayer case. They reproduce the hexagonal lattice symmetry of the monolayer in the normal state, with contrast primarily arising from the Se sublattice. This hexagonal pattern is additionally modulated by the Au(111) substrate, leading to a moiré pattern with an emergent length scale that depends on the angle between Au(111) and monolayer VSe_2_ (Figure 4(b)).

**Figure 4:**
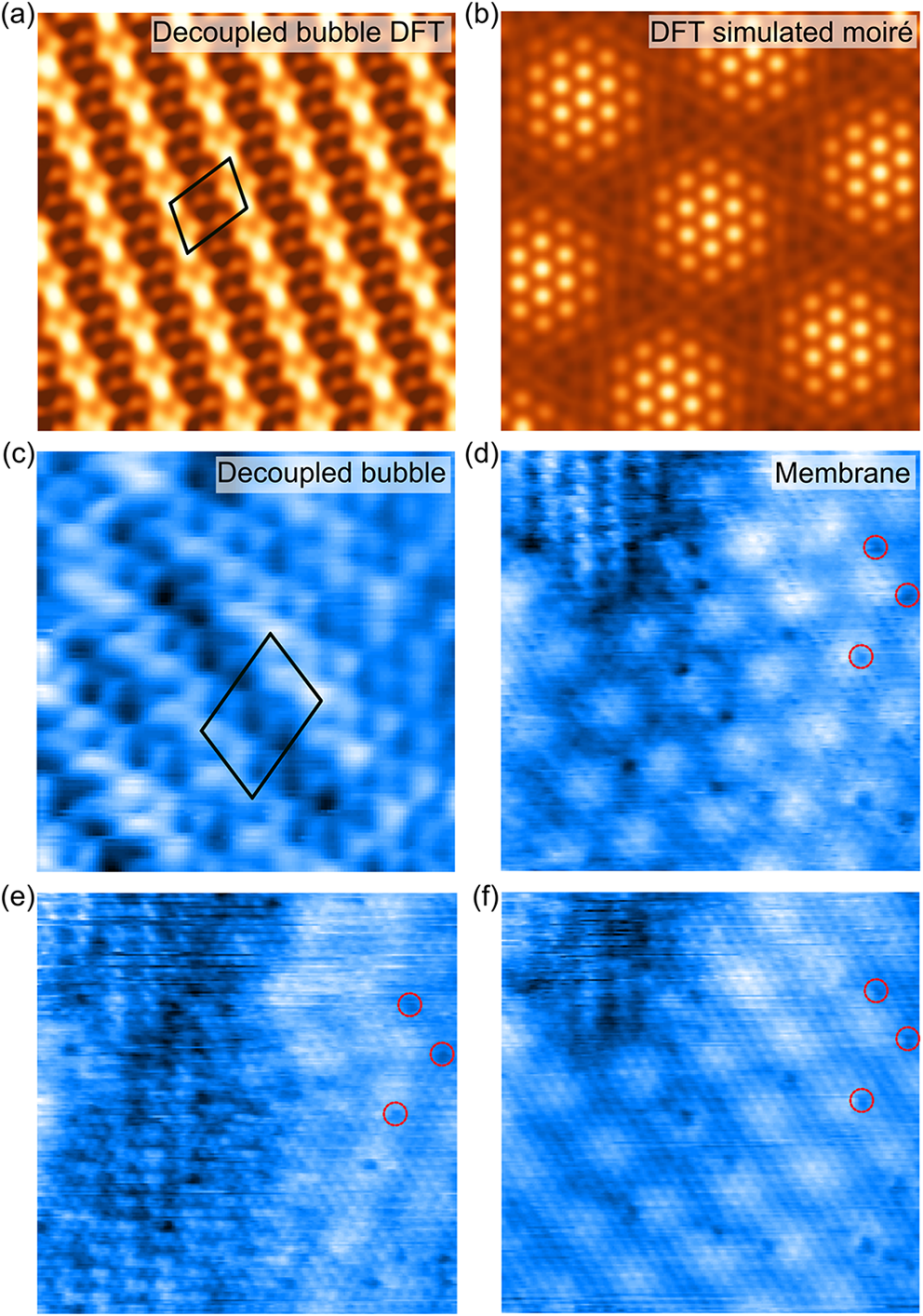
(a) Simulated STM images (5 × 5 nm^2^) of a decoupled VSe_2_ ML displaying a √3*a* × √7*a* CDW phase (supercell displayed in black), and (b) of a coupled VSe_2_ ML showing a moiré pattern rather than a CDW. (c) STM topography of the √3*a* × √7*a* ML-CDW developing on ML bubbles (3 × 3 nm^2^, *V*_bias_ = 200 mV, *I*_t_ = 50 pA). (d–f) Consecutive STM topographic images of the same area of a ML (see the three defects highlighted in red as references). They show a decoupled ML membrane with its ML-CDW surrounded by moiré regions where the ML is coupled to the gold substrate. It shows the reversible coupling and decoupling of the membrane to the substrate in the lower left region. (10 × 10 nm^2^, −200 mV, 1 nA).

### Membranes and Bubbles of Monolayer VSe_2_ on Au(111)

During the exfoliation process, due to trapped residues between the flake and the substrate, bubbles can form (see, e.g., areas marked with orange circles in Figure 1(a) and Supporting Figure 1). Meanwhile, at other locations, the flakes can form membranes suspended over pits naturally present on the gold terraces (Supporting Figure 2). Our samples host many of these structures. Bubbles appear as bright dome-shaped areas that stand out against the flat background of VSe_2_ (Figure 1(a) and Supporting Figures 1 and 8). Conversely, membranes appear flat or even slightly lower than the surrounding VSe_2_ ML (Figure 4(d–f)). Both are identifiable by the absence of moiré pattern in their high-resolution STM imaging in Figure 4. They instead reveal a √3*a* × √7*a* periodic modulation in topographic STM images, consistent with the monolayer CDW (ML-CDW) previously reported for MLs^21,23,24,26^ and with our DFT calculations for this CDW phase in Figure 4(a).

Figure 4(d)–(f) are particularly revealing of the effect of coupling a ML to the gold substrate. They show a decoupled membrane with its √3*a* × √7*a* CDW surrounded by the moiré pattern characteristic of a coupled ML. When repeatedly scanning the same area using a high current set point where the tip/sample interaction is strong, we observe a reversible switching between the moiré and the ML-CDW in the lower left-hand region of the image. We interpret this as a region where the ML alternates between coupled and decoupled states, providing direct experimental evidence that the interaction with the substrate is responsible for quenching the CDW phase.

Coupled ML and BL areas consistently show a metallic spectrum with a marked peak below the Fermi level (*V*_bias_ = 0 V). The precise position of this peak depends on thickness, with a shift to lower energies in the BL regions (Figure 5(a)). Similar spectra are observed for all coupled monolayer regions independent of their distinct moiré patterns and twist angles. Although minor variations in the peak position are present, they do not evolve systematically with twist angle and remain much smaller than the spectroscopic contrast observed between coupled and decoupled monolayers (Supporting Figure 8­(b,c)). These observations are very well reproduced by our DFT calculated DOS in Figure 5(c).

**Figure 5:**
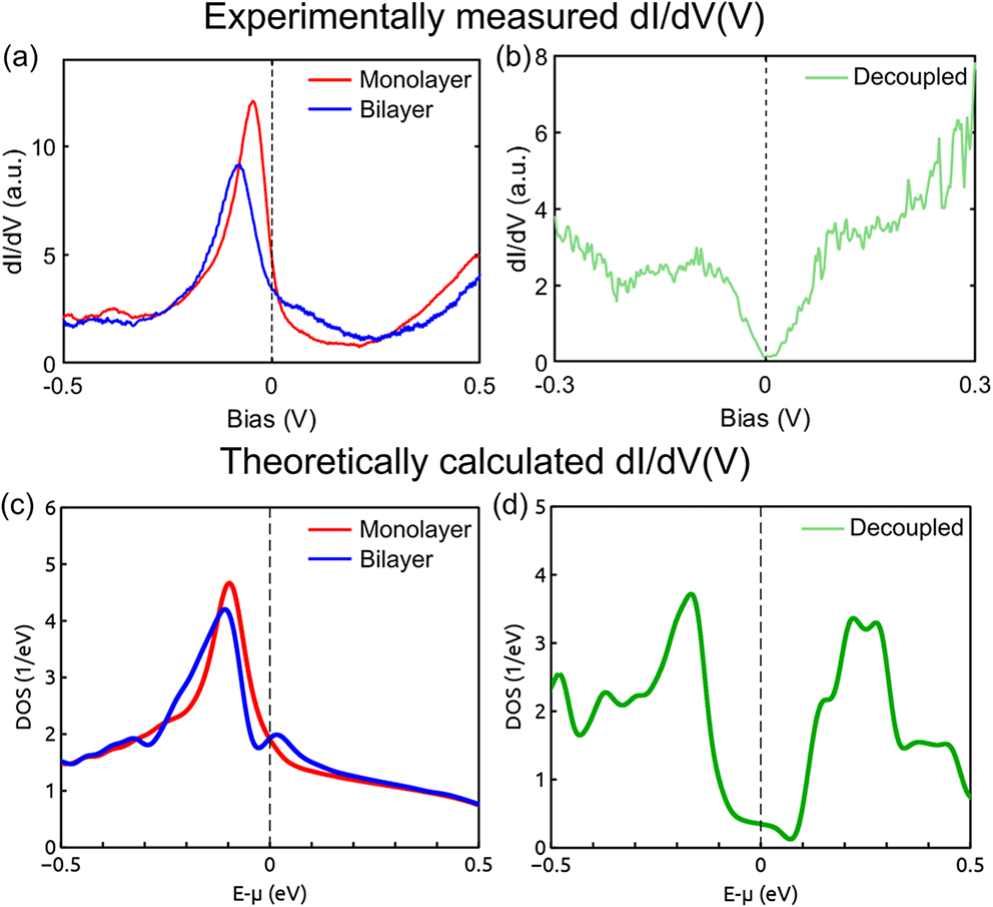
(a) Differential tunneling conductance spectra measured on a coupled monolayer with a moiré pattern and a bilayer with a 4*a* × 4*a* CDW. (b) Differential tunneling conductance spectra measured on a decoupled monolayer with a √3*a* × √7*a* CDW. (c, d) Show the corresponding DFT model calculations.

Decoupled areas with the ML-CDW show more variability than coupled ML in their spectroscopic signatures, ranging from a slightly suppressed peak near the Fermi level to an entirely gapped spectrum, as shown in Figure 5(b). The latter is consistent with our calculations for a decoupled ML as shown in Figure 5(d). Proximity effects may induce shifts in the chemical potential, while the local density of states can vary depending on the measurement site within the CDW supercell, as discussed in Supporting Information Section VI. This variability likely reflects local differences in the degree of decoupling and in the geometry of the suspended regions, but in all cases the spectra are clearly distinct from those of the coupled monolayer. This behavior is consistent with the broad diversity of low-energy spectral responses reported previously for monolayer VSe_2_ on other substrates.^20,22–24,26,29^ These bubble and membrane nanostructures have been observed in other TMDs and were found to be electronically decoupled from the substrate.^6,53,54^ This distinction is further supported by spatially resolved conductance maps at different energies, where the decoupled bubbles exhibit a reduced DOS compared to the surrounding coupled region, as described in Supporting Information Section V.

## Discussion

Exfoliated VSe_2_ flakes have been reported to maintain a 4*a* × 4*a* CDW modulation down to three layers,^1^ however with a transition temperature depending on thickness. The CDW first weakens in thin layers, but strengthens again in the thinnest layers, as a result of dimensional crossover and confinement effects. Our observations for the BL flakes are consistent with these observations, as they still exhibit the 4*a* × 4*a* CDW modulation when exfoliated on gold. The bottom layer acts as a decoupling layer for the top layer of BL VSe_2_. This enables the formation of the 4*a* × 4*a* CDW in the top layer. This is in agreement with previous observations reporting that BL VSe_2_ exhibits a 4*a* × 4*a* CDW modulation when grown on less metallic substrates such as bilayer graphene^29^ and graphite.^32^

In our exfoliated ML flakes on Au, we access a substrate-dominated regime where interfacial hybridization and charge transfer become key control parameters of the CDW. In ML VSe_2_ flakes coupled to gold, the interaction with the substrate completely dominates the CDW behavior and the latter is entirely suppressed, as revealed by our experiments and calculations. In addition, ML VSe_2_ on gold reveals pronounced metallic behavior, characterized by a strong peak in the DOS near the Fermi level in perfect agreement with theory. When the coupling to the substrate is removed, as in the case of strained bubbles and suspended membrane-like flakes, the √3*a* × √7*a* CDW emerges, consistent with the absence of hybridization for free-standing ML VSe_2_ and a tensile regime.

As previously discussed, the suppression of CDW may lead to the emergence of magnetic ordering^37,38,55^ in exfoliated ML VSe_2_ flakes on gold. The non spin-polarized calculations presented in this work reproduce all the main features found in our experiments. However, they do not exclude the emergence of magnetism in the ML limit, specifically at the boundary between coupled and decoupled regions with the gold substrate. Since the STM measurements performed here do not provide direct insight into the spin texture of the flakes, another experimental probe is needed to assess the magnetic properties. Optical means (e.g magneto-optical Kerr rotation) to probe these 2D layers are not suitable due to the lack of an in-plane electric field component on the metallic substrate. Other scanning probe techniques, such as magnetic force microscopy (MFM) and scanning SQUID microscopy (SSM), are promising alternatives. Our experimental setup does not provide access to these techniques in the same UHV system as the STM. Therefore, the samples must be transferred, and their surface must be protected. Due to these technical challenges, our first attempts with these techniques do not yet allow us to firmly conclude on the magnetic properties, and they call for further experimental efforts to probe the possible magnetic texture at the interface between ML VSe_2_ and the gold.

## Conclusion

In conclusion, we investigated both ML and BL VSe_2_ flakes on a gold substrate, accompanied by theoretical modeling. Our results show that BLs largely retain their bulk-like character and exhibit a 4*a* × 4*a* CDW order, whereas MLs are strongly coupled to the substrate, displaying only a moiré supermodulation without CDW formation. When MLs become decoupled from the gold surface, in strained bubbles and suspended membranes, the √3*a* × √7*a* CDW modulation emerges, highlighting the decisive role of interfacial hybridization and charge transfer in suppressing CDW order. Altogether, our findings identify exfoliated VSe_2_ on gold as a promising platform for investigating the subtle interplay between competing charge-order phases in two dimensions through interface hybridization.

## Methods

### Sample Preparation

Commercial VSe_2_ single crystals (HQ Graphene) were exfoliated using a slightly modified version of the gold-assisted exfoliation protocol described in ref 6. Instead of performing the entire exfoliation in a glovebox, we placed a large freshly cleaved bulk VSe_2_ crystal on a freshly exposed template-stripped gold substrate,^6^ performing the final exfoliation inside the UHV system using the wheel setup described in ref 56. This procedure avoids exposing the surface to atmosphere. No further *in situ* treatment of the sample was done.

### STM/STS Characterization

The scanning tunneling microscopy (STM) and spectroscopy (STS) experiments were performed with a Specs JT Tyto STM at 4.5 K, at a base pressure better than 1 × 10^–10^ mBar. We used electrochemically etched tungsten or iridium tips, which were cleaned *in situ* by Ar^+^ ion sputtering before conditioning and characterizing on a Au(111) single crystal. STM topographic images were recorded in constant current mode. The d*I*/d*V*(*V*) conductance spectra were acquired using a lock-in amplifier with a sample bias modulation amplitude in the range of 2 to 10 mV at 347 Hz.

### DFT and Numerical Calculations

We have performed *ab initio* electronic structure calculations based on density functional theory (DFT)^57,58^ for monolayer VSe_2_ in both the free-standing and Au(111)-coupled configurations. Calculations were carried out using the plane-wave pseudopotential method as implemented in the Quantum ESPRESSO package.^59,60^ The generalized gradient approximation in the Perdew–Burke–Ernzerhof (GGA-PBE) scheme was employed for the exchange–correlation functional.^61^ Core–valence interactions were described using standard ultrasoft pseudopotentials from the PSLibrary.^62^

The kinetic energy cutoff for the plane-wave basis was set to 60 Ry for the wave functions and 700 Ry for the charge density. Brillouin zone integrations were performed using a 12 × 12 × 1 Monkhorst–Pack *k*-point mesh. A small electronic smearing of 0.01 Ry was used in all calculations. A vacuum spacing of 20 Å was included along the out-of-plane direction to avoid spurious interactions between periodic replicas.

For the coupled configuration, the VSe_2_ monolayer was placed on a commensurate Au(111) slab constructed from the experimental lattice constant of Au. Importantly, a minimum thickness of six gold layers in the (111) direction was required to capture the spectral behavior observed in the experiments accurately. To reduce computational cost while maintaining accuracy, the bottom layers of the Au slab were kept fixed during relaxation, while all V and Se atoms and the topmost Au layers were allowed to relax. Atomic structures were relaxed until the forces on each atom were smaller than 10^–4^ Ry/Bohr, and the total energy convergence threshold was set to 10^–9^ Ry. For the √3*a* × √7*a* reconstruction, the initial structural configuration was obtained by displacing the atoms along the eigenvector of the unstable phonon mode associated with the CDW instability, which facilitates convergence toward the distorted phase. The stabilization of this phase follows the procedure discussed in ref 40.

Harmonic phonon spectra were obtained using density functional perturbation theory (DFPT), as implemented in Quantum ESPRESSO. The dynamical matrices were computed on a uniform 8 × 8 × 1 *q*-point grid and subsequently Fourier-interpolated to obtain the phonon band structures. We note that the phonon spectrum is sensitive to the electronic smearing: increasing the smearing suppresses the imaginary phonon modes, while smaller values enhance the dynamical instabilities associated with the CDW phases.

## Supplementary Material



## Notes

Large scale scanning probe characterization of the sample, bias-dependent STM imaging of the moiré pattern, distinction between moiré and CDW modulations, Fourier transforms of the different superstructures observed in VSe_2_ on gold, spatially resolved spectroscopy maps on coupled and decoupled VSe_2_ monolayers, calculated local density of states in the √3*a* × √7*a* CDW phase (PDF)
