# Supplementary material for: Tuning Competing Electronic Phases in Monolayer VSe2 via Interface Hybridization
Source: ACS Nano. 2026 Jul 24;20(31):21767–74. doi: 10.1021/acsnano.6c04065 (PMC13472204; doi:10.1021/acsnano.6c04065)
Supplement: Supplementary file 1 [file nn6c04065_si_001.pdf]

Supporting Information for Tuning competing electronic phases in monolayer VSe_2 via interface hybridization

Ishita Pushkarna[†,1] Árpád Pásztor[†,1] Greta Lupi,[2]
Adolfo O. Fumega,[2] and Christoph Renner[*1]

[1] *Department of Quantum Matter Physics, University of Geneva, 1211 Geneva, Switzerland*

[2] *Department of Applied Physics, Aalto University, 02150 Espoo, Finland*

[†] *These authors contributed equally to this work.*

[*] *Corresponding Author: Orcid.org/0000-0001-9882-681X; Email: christoph.renner@unige.ch*

CONTENTS

I. Large-scale characterization of the sample 2

II. Bias-dependent STM imaging of the moiré pattern 4

III. Uniformity of moiré amplitude versus spatial variation of CDW 8

IV. FFTs of different superstructures observed in VSe_2 on gold 9

V. Spatially resolved spectroscopy maps on coupled and decoupled VSe_2 monolayers 11

VI. Local density of states in the $\sqrt{3}a \times \sqrt{7}a$ charge density wave phase 12

References 13

I. LARGE-SCALE CHARACTERIZATION OF THE SAMPLE

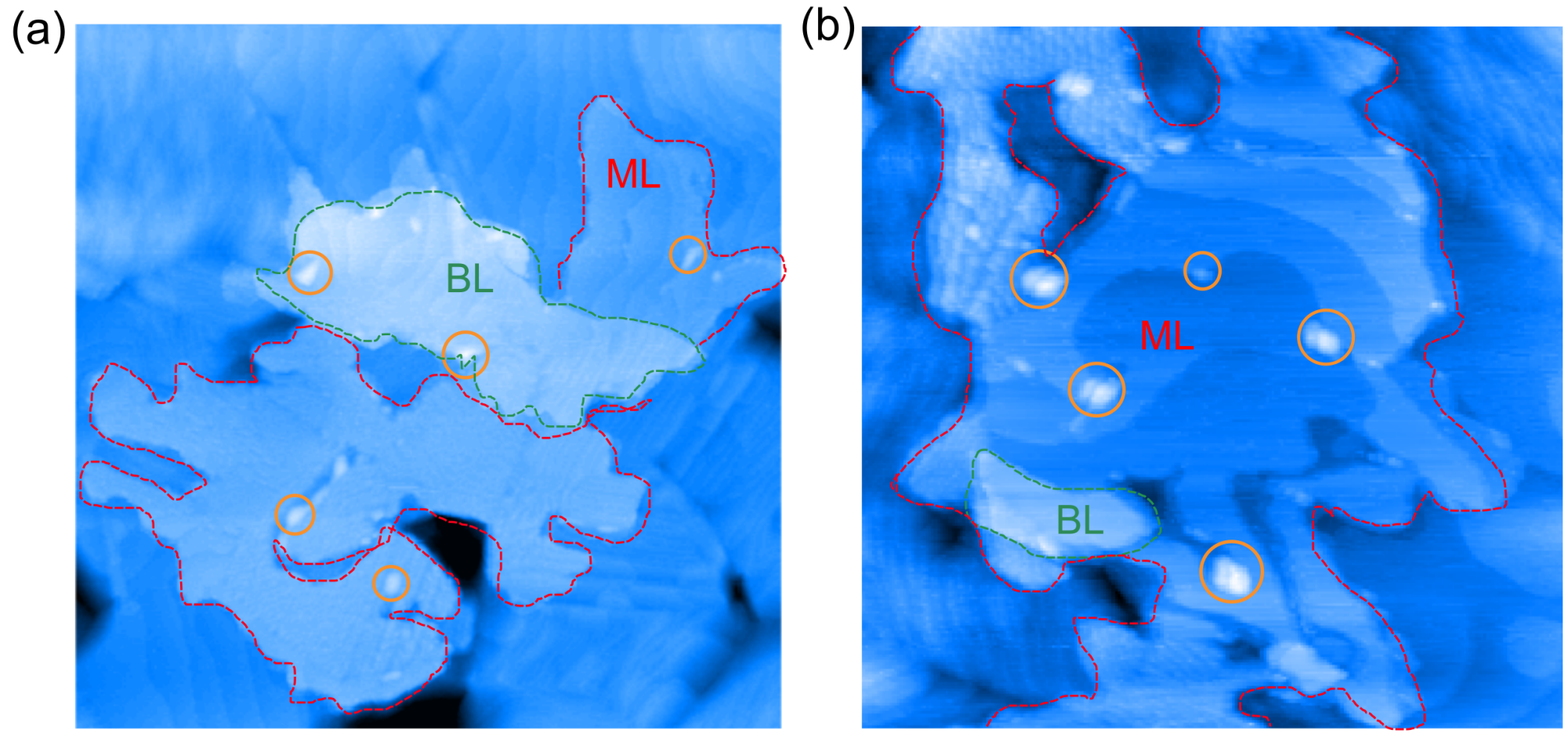

Supporting Figure 1. (a) 400 × 400 nm^2 and (b) 250 × 250 nm^2 STM topographies highlighting different morphologies that we observe in our exfoliated sample. These include ML flakes (contour marked in red), BL flakes (contour marked in green), and decoupled bubbles (circled in orange). Scanning parameters are (a) V_{bias} = 500 mV, I_t = 40 pA, (b) 500 mV, 50 pA.

Supporting Figure 1 shows two representative examples of large-scale characterization of exfoliated VSe_2 on gold. As discussed in the main text, exfoliation predominantly yields monolayer (ML) flakes, along with a smaller fraction of bilayer (BL) regions and locally decoupled bubbles.

Occasionally, the gold surface exhibits shallow depressions. During exfoliation, flakes can span these pits, resulting in suspended, membrane-like regions. In Supporting Figure 2, we show a few examples of pits in the bare gold substrate where such suspended flakes can be found.

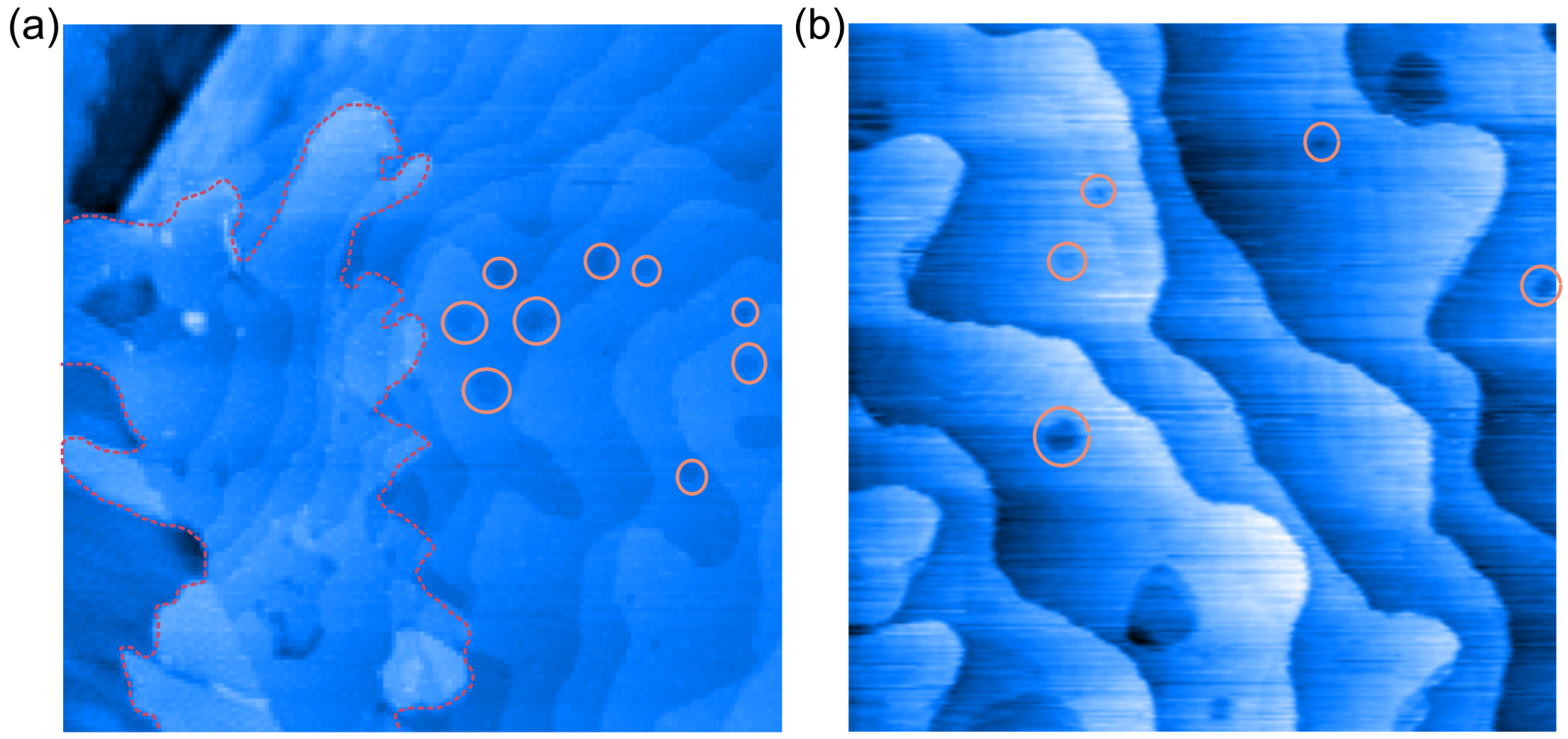

Supporting Figure 2. (a) 300 $\times$ 300 nm^2 STM image with highlighted ML (red contour) and flat gold terraces with small pits (in orange). (b) 150 $\times$ 150 nm^2 STM image taken on gold terraces next to an exfoliated flake, showing a faint herringbone superstructure (as observed on Au(111)) and the presence of pits of various sizes and depths. Scanning parameters: (a) V_{bias} = 500 mV, I_t = 50 pA, (b) 5 mV, 5 nA.

II. BIAS-DEPENDENT STM IMAGING OF THE MOIRÉ PATTERN

A decisive confirmation that the observed superstructure originates from moiré patterns rather than a CDW is provided by bias-dependent STM imaging. To compare the superstructure observed in ML and bulk VSe_2, we characterized a cleaved-bulk VSe_2 crystal, which is reported to show a $4a \times 4a$ CDW modulation [1]. As shown in Supporting Figure 3, the CDW modulation exhibits a characteristic contrast inversion when the bias is swept across the CDW gap. This can be visualized from the defect (marked by a red circle) at the bottom of the images, which lies at a CDW minimum in panels (a) and (b) when the imaging bias is negative. However, in panels (c) and (d), CDW contrast inverts with respect to the negative bias, and now the defect sits on a maximum of the CDW periodicity. This contrast inversion across the CDW gap is a characteristic of the CDW order [2, 3].

In contrast to the bulk VSe_2, our measurements on exfoliated ML VSe_2 reveal no such contrast inversion for the superstructure. This is illustrated in Supporting Figures 4 and 5, showing STM images of the moiré pattern for a twist angle of 2.5°, where both the VSe_2 atomic lattice and the superimposed moiré modulation are clearly resolved at different biases. All images were obtained over the same scan area, and the defects clearly show there is no contrast inversion for all bias voltages considered here.

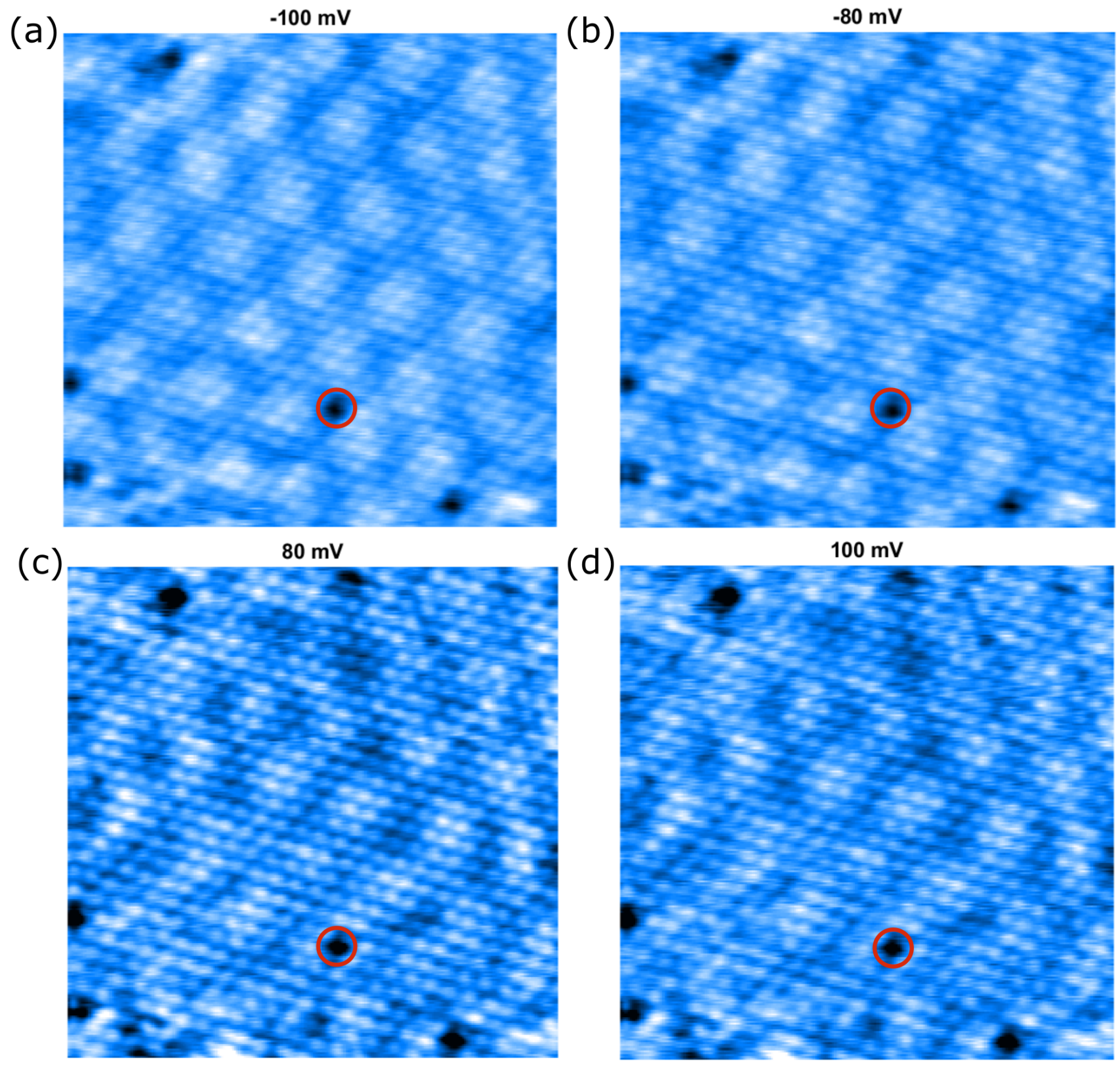

Supporting Figure 3. Bias-dependent CDW imaging of the same 8×8 nm^2 area on cleaved bulk VSe_2. Set points are: (a) V_{bias} = -100 mV, I_t = 200 pA, (b) -80 mV, 200 pA, (c) 80 mV, 200 pA, (d) 100 mV, 200 pA. CDW contrast is inverting when going from negative to positive bias polarity.

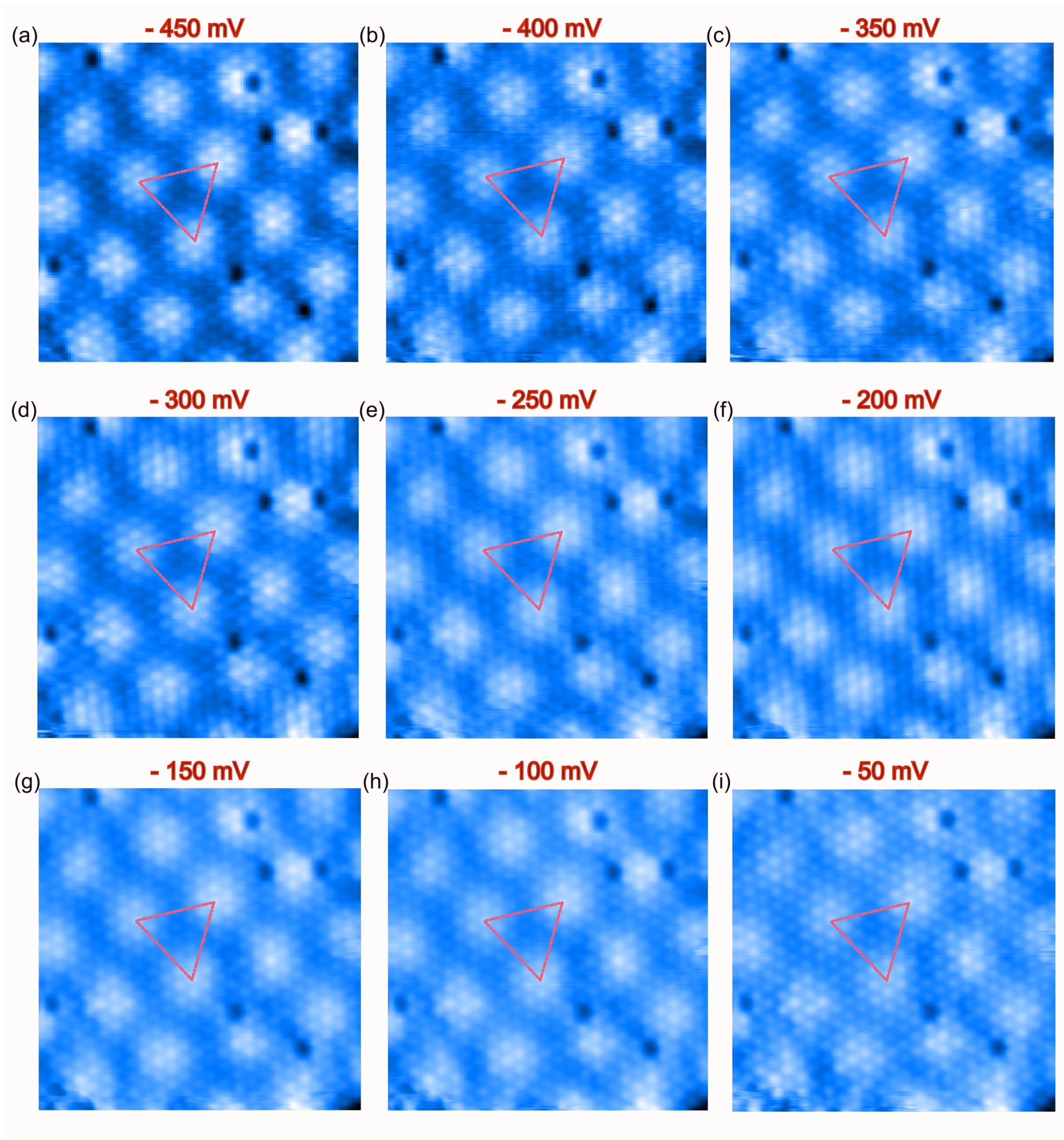

Supporting Figure 4. Bias-dependent 7×7 nm^2 STM images from -450 mV to -50 mV of the moiré superstructure observed on ML VSe_2 on Au(111). The same overlaid red triangle connects three moiré maxima in all images, confirming that the moiré pattern does not shift as a function of bias.

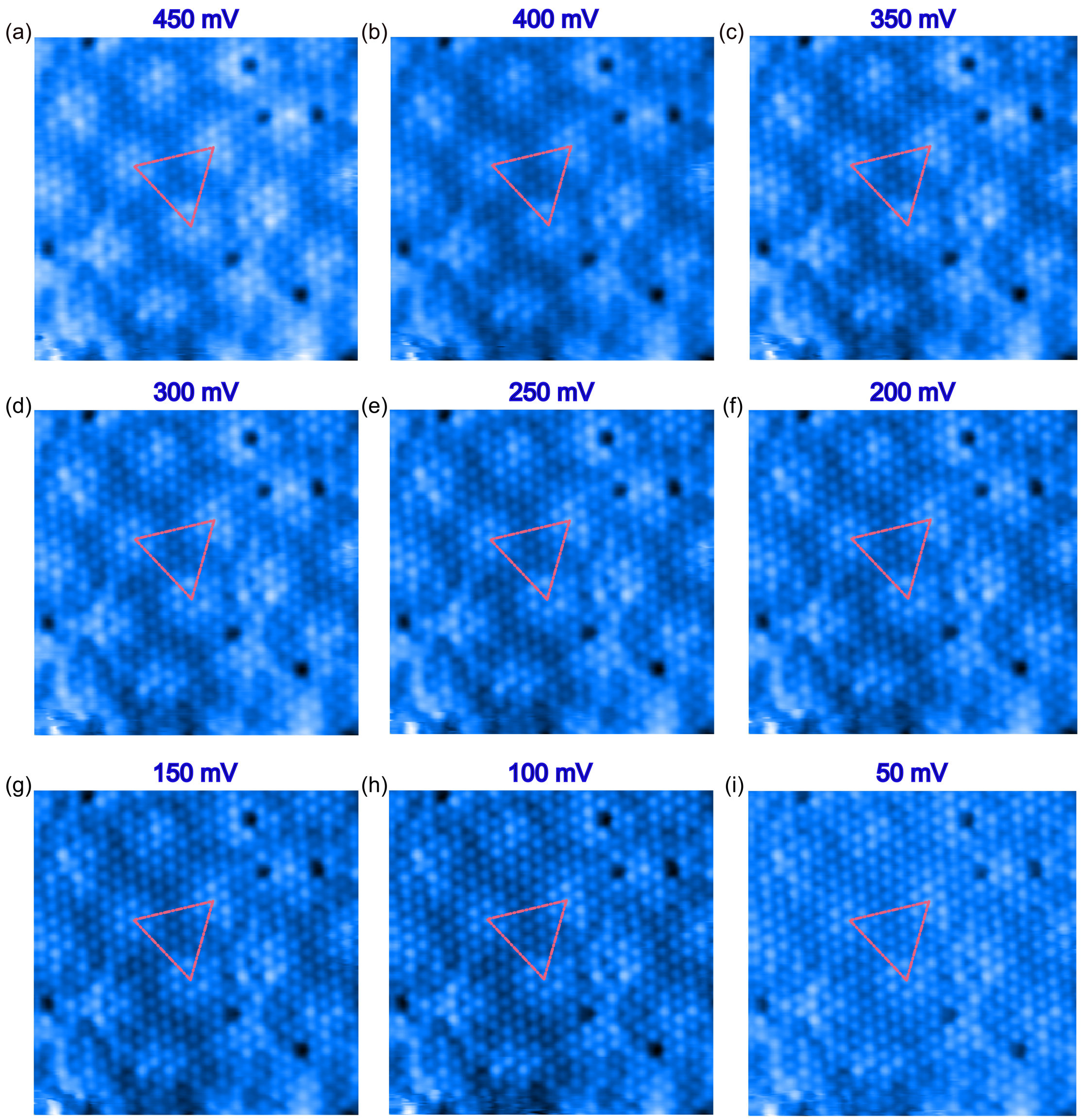

Supporting Figure 5. Bias-dependent 7×7 nm^2 STM images from +50 mV to +450 mV of the moiré superstructure observed on ML VSe_2 on Au(111) over the same area as in Supporting Figure 4. The same overlaid red triangle as in Supporting Figure 4 connects three moiré maxima in all images, confirming that the moiré pattern does not shift as a function of bias and polarity.

III. UNIFORMITY OF MOIRÉ AMPLITUDE VERSUS SPATIAL VARIATION OF CDW

It has been reported that the amplitude of each CDW component can vary from one region to another (in the same scan direction), even when there is no tip change [4]. This is another clear feature that can be used to distinguish CDWs from moiré patterns. In the dataset discussed here, we observe that the amplitude of the moiré pattern remains uniform in all directions, giving rise to a symmetric modulation across spatial locations and over the entire bias range. Moreover, while the CDW shows weak pinning around defect sites [4], the moiré pattern persists as a continuous and undisturbed modulation, unaffected by defects (Supporting Figures 4 and 5). Finally, the CDW lattice is aligned and commensurate with the atomic lattice in VSe_2 [4] (Supporting Figure 6), which is not the case for the moiré pattern (Supporting Figure 7). These features further confirm that the modulation observed here is a moiré pattern rather than a CDW.

IV. FFTS OF DIFFERENT SUPERSTRUCTURES OBSERVED IN VSE_2 ON GOLD

The FFT analysis provides reciprocal-space support for the real-space assignment of the observed superstructures. CDW modulations produce additional peaks at commensurate positions relative to the VSe_2 atomic lattice, independent of tunneling bias voltage, as observed for the $4a\times4a$ modulation in bulk and bilayer VSe_2 and for the $\sqrt{3}a\times\sqrt{7}a$ modulation in decoupled monolayer regions (see Supporting Figure 6). By contrast, the superstructure peaks in coupled monolayer VSe_2 shift in position and orientation depending on the local Au(111) substrate orientation, while the VSe_2 atomic lattice peaks remain unchanged (see Supporting Figure 7). This behavior is characteristic of a moiré pattern generated between a given VSe_2 flake and a Au(111) substrate with different local twist angles, and distinguishes it from the CDW modulations.

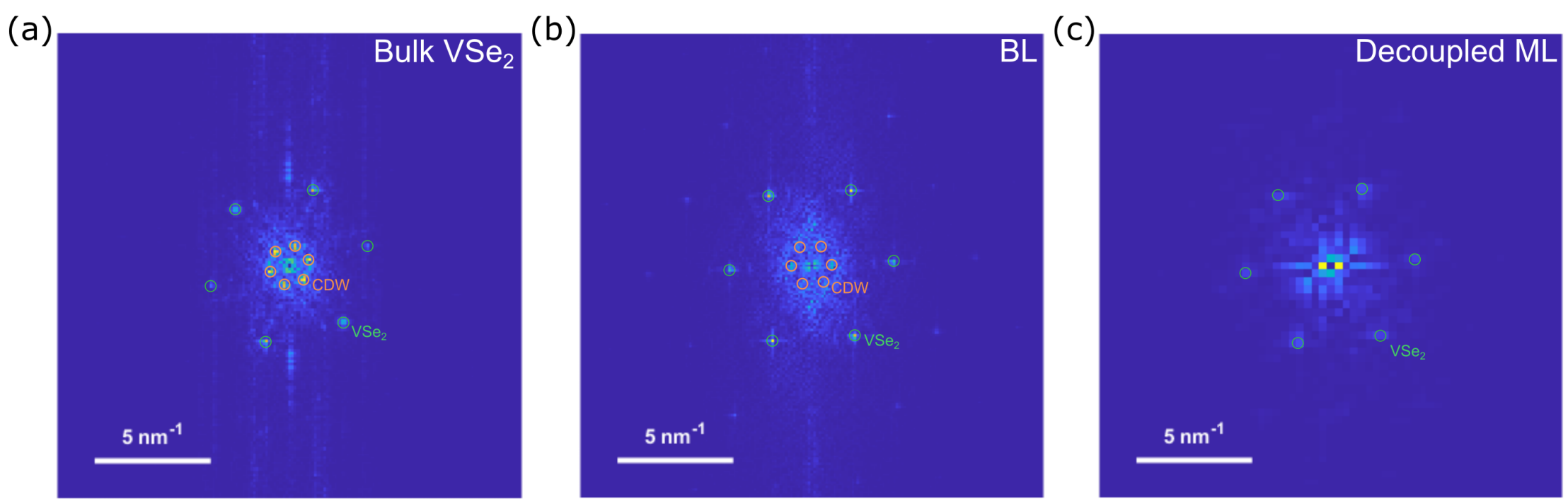

Supporting Figure 6. FFTs of the CDW modulations observed in (a) bulk VSe_2, (b) exfoliated bilayer VSe_2 on Au(111), and (c) a decoupled monolayer VSe_2 region. The atomic lattice peaks are highlighted by green circles, while the commensurate CDW peaks are highlighted by orange circles. In panels (a) and (b), the additional peaks correspond to the $4a \times 4a$ CDW modulation, consistent with the bulk-like CDW order observed in bilayer VSe_2. In panel (c), the additional peaks correspond to the $\sqrt{3}a \times \sqrt{7}a$ modulation observed in decoupled monolayer VSe_2.

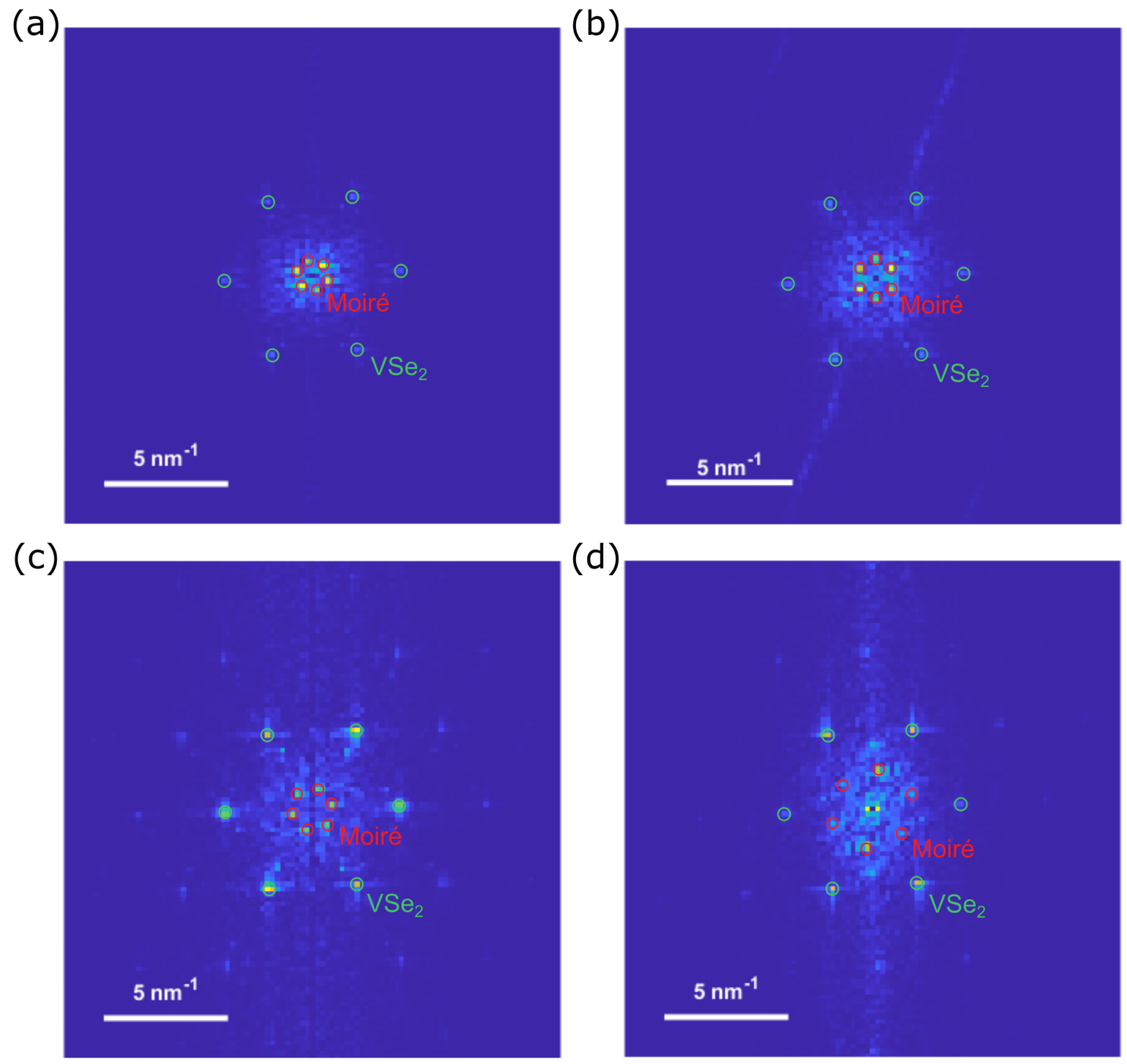

Supporting Figure 7. FFTs corresponding to the different moiré patterns observed in exfoliated monolayer VSe_2 on Au(111). The panels are presented in the same order as in Figure 3 of the main text. The VSe_2 atomic lattice peaks are highlighted by green circles, while the moiré peaks are highlighted by red circles. Note that these peaks change position depending on the local twist angle between the monolayer VSe_2 and the underlying Au(111) grain (whereas the VSe_2 peaks remain unchanged), clearly identifying them as moiré peaks and excluding a CDW origin.

V. SPATIALLY RESOLVED SPECTROSCOPY MAPS ON COUPLED AND DECOUPLED VSE_2 MONOLAYERS

Supporting Figure 8 shows three Au(111) terraces covered with a VSe_2 ML and a large bubble. The bubble appears high (bright contrast) in the topography (panel (a)). The two conductance maps in panels (b) and (c) show a distinctive lower DOS in the decoupled bubble region compared with the surrounding ML, closely following the Au(111) terraces. The corresponding detailed tunneling spectra are shown in Figure 5(a) and (b) in the main text: the regions where the ML follows the Au(111) substrate, and where high-resolution topography shows a moiré pattern, exhibit a conductance peak near E_F. The bubble region where the ML is decoupled from the Au(111) substrate, and where high resolution topography shows the $\sqrt{3}a \times \sqrt{7}a$ CDW, exhibits a strongly reduced DOS at low energy.

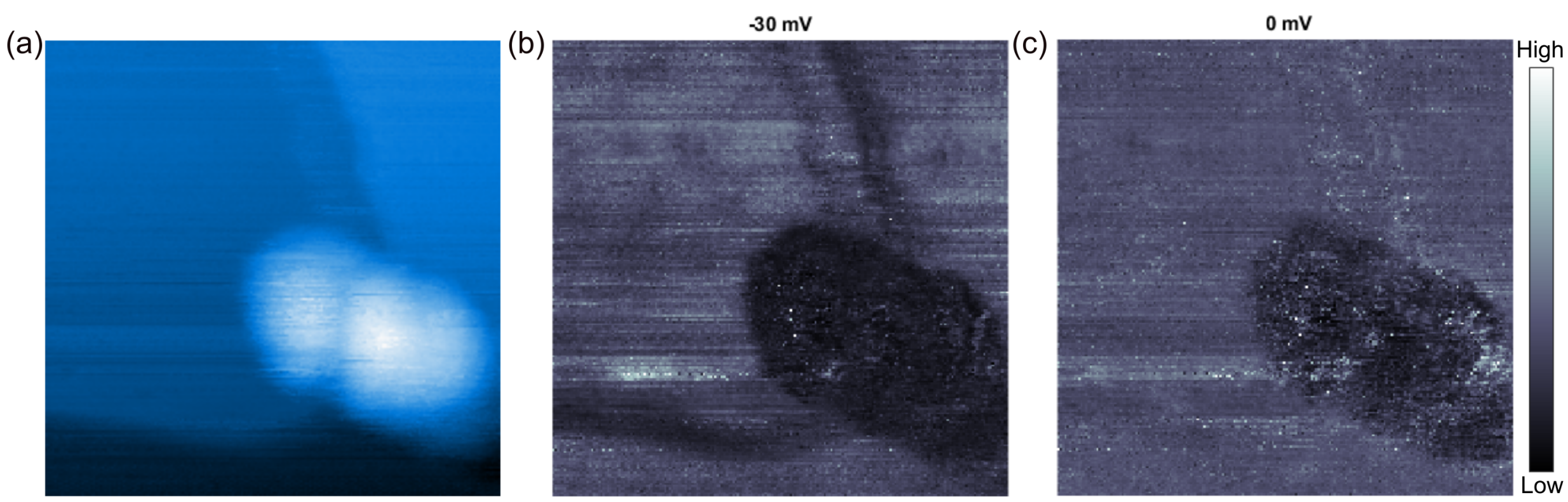

Supporting Figure 8. (a) 20×20 nm^2 STM topography acquired simultaneously with the $dI/dV(V,\vec{r})$ map over a ML region crossing several gold steps, with a prominent decoupled bubble in the lower right region. (b), (c) Conductance map slices taken from the $dI/dV(V,\vec{r})$ map at -30 mV and 0 mV, respectively. Set point during the conductance map: V_{bias}= 100 mV; I_t= 300 pA.

VI. LOCAL DENSITY OF STATES IN THE $\sqrt{3}a \times \sqrt{7}a$ CHARGE DENSITY WAVE PHASE

To further assess the relationship between the calculated electronic structure and the tunneling spectra measured by STM, we compute the local density of states (LDOS) at the inequivalent V sites of the $\sqrt{3}a \times \sqrt{7}a$ CDW supercell. While the density of states shown in the main text corresponds to the total DOS obtained from density functional theory, STM probes a local quantity whose spectral shape depends on the specific atomic environment beneath the tip.

Supporting Figure 9 shows the LDOS projected onto the three inequivalent V atoms within the CDW supercell. All sites exhibit a pronounced suppression of spectral weight around the Fermi energy, consistent with the formation of the CDW phase. However, significant differences are observed in the detailed spectral lineshape. In particular, the relative positions and intensities of the spectral maxima vary between inequivalent sites, reflecting the modulation of the local electronic structure induced by the CDW reconstruction.

These results highlight that the tunneling spectrum measured experimentally may depend on the precise location of the STM tip within the CDW unit cell. Consequently, the measured dI/dV spectrum does not necessarily correspond directly to the total DOS, but rather to a local probe of the electronic structure that may emphasize the contribution of specific atomic environments. Furthermore, experimental spectra can also reflect an average over several inequivalent regions within the probed area.

We note that the experimentally observed minimum in the dI/dV spectrum is centered at the chemical potential, whereas the minimum in the calculated DOS appears slightly shifted. Such a difference may arise from residual interactions between the nominally decoupled monolayer and its environment, including weak charge transfer from the substrate or trapped residues. These effects are not explicitly included in the present calculations and can lead to small shifts of the chemical potential without substantially modifying the overall electronic structure associated with the CDW phase. Therefore, the most robust feature for comparison between theory and experiment is the characteristic CDW-induced suppression of low-energy spectral weight, which is consistently reproduced in both cases.

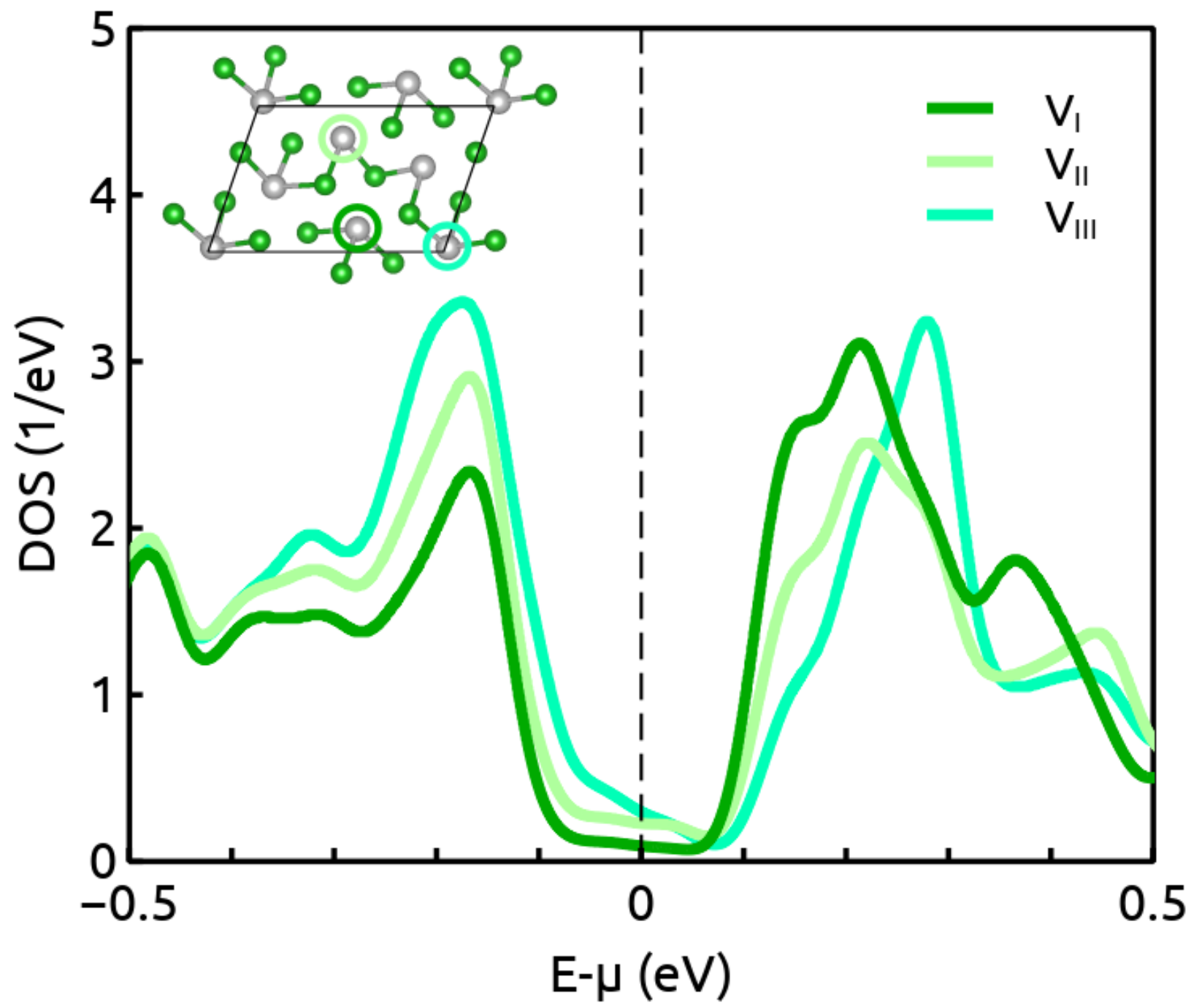

Supporting Figure 9. Local density of states projected onto the three inequivalent V atoms of the $\sqrt{3}a \times \sqrt{7}a$ CDW supercell of monolayer VSe_2. While all sites display a suppression of low-energy spectral weight associated with the CDW phase, the detailed spectral lineshape varies depending on the local atomic environment.

---

[1] Pásztor, A. Scarfato, C. Barreteau, E. Giannini, and C. Renner, "Dimensional crossover of the charge density wave transition in thin exfoliated VSe_2," *2D Materials*, vol. 4, p. 041005, Sept. 2017. Publisher: IOP Publishing.

[2] Pásztor, A. Scarfato, M. Spera, F. Flicker, C. Barreteau, E. Giannini, J. v. Wezel, and C. Renner, "Multiband charge density wave exposed in a transition metal dichalcogenide," *Nature Communications*, vol. 12, p. 6037, Oct. 2021. Publisher: Nature Publishing Group.

[3] M. Spera, A. Scarfato, A. Pásztor, E. Giannini, D. R. Bowler, and C. Renner, "Insight into the charge density wave gap from contrast inversion in topographic STM images," *Physical Review Letters*, vol. 125, no. 26, p. 267603, 2020.

[4] Á. Pásztor, A. Scarfato, M. Spera, C. Barreteau, E. Giannini, and C. Renner, "Holographic

imaging of the complex charge density wave order parameter," *Physical Review Research*, vol. 1, no. 3, p. 033114, 2019.
